# Comparative evaluation for small molecule somatostatin 4 receptor agonists: *in silico*, *in vitro*, and *in vivo* approaches

**DOI:** 10.3389/fphar.2026.1838782

**Published:** 2026-06-15

**Authors:** Dóra Biskup, Minjin Ganbold, Andrea Kinga Nehr-Majoros, Valéria Tékus, Zsófia Hajna, Barbara Fülöp, Éva Szőke, Csaba Hetényi, A. Michael Crider, Iman Daryaei, Zsuzsanna Helyes, Rita Börzsei, Erika Pintér

**Affiliations:** 1 Department of Pharmacology and Pharmacotherapy, Medical School & Centre for Neuroscience, University of Pécs, Pécs, Hungary; 2 National Laboratory for Drug Research and Development, Budapest, Hungary; 3 Hungarian Research Network, Chronic Pain Research Group, Pécs, Hungary; 4 PharmInVivo Ltd., Pécs, Hungary; 5 Department of Laboratory Diagnostics, Faculty of Health Sciences, University of Pécs, Pécs, Hungary; 6 Algonist Biotechnologies GmbH, Vienna, Austria; 7 Eötvös Lorand Research Network, Chronic Pain Research Group, University of Pécs, Pécs, Hungary; 8 Department of Pharmaceutical Sciences, School of Pharmacy, Southern Illinois University Edwardsville, Edwardsville, IL, United States; 9 Department of Chemistry and Biochemistry, University of Arizona, Tucson, AZ, United States

**Keywords:** cAMP assay, docking, hyperalgesia, neuropathic pain, somatostatin receptor subtype 4

## Abstract

**Introduction:**

The treatment of neuropathic pain with traditional and adjuvant analgesics is limited in efficacy and associated with severe adverse effects. Our group has previously shown that somatostatin, released from capsaicin-sensitive peptidergic sensory nerve terminals, mediates analgesic and anti-inflammatory effects at the peripheral and central levels via the somatostatin receptor subtype 4 (SST4). The therapeutic use of native somatostatin is limited by its numerous biological actions mediated by the five somatostatin receptors and its short elimination half-life. Therefore, the development of SST4-selective agonists may be an effective approach for treating neuropathic pain.

**Methods:**

In this comparative study, the receptor binding and activation properties of four small molecules were compared with those of somatostatin and the SST4 receptor superagonist, J-2156, through in silico drug-likeness investigation, pharmacokinetic prediction, molecular docking calculations, in vitro cAMP accumulation assays, and in vivo assessment of their antihyperalgesic effects in the partial sciatic nerve ligation mouse model of traumatic neuropathy.

**Results and Discussion:**

Docking calculations showed that all compounds interact with the conserved Asp126 of SST4, which is suggested to play a key role in ligand binding. cAMP assays confirmed that all molecules activate the SST4 receptor with comparable potency to that of J-2156. All examined compounds reversed PSNL-induced mechanical hyperalgesia. Their antihyperalgesic effects ranged from 20% to 70% and depended on both the time and the applied dose. These results suggest that the tested compounds could be potentially effective in the treatment of neuropathic pain.

## 1 Introduction

Somatostatin (SRIF-14) is prevalent in the central and peripheral nervous systems, as well as in various peripheral tissues and immune cells. It inhibits the release of numerous neurotransmitters and hormones in both the CNS and periphery, including growth hormone, thyroid-stimulating hormone, GABA, dopamine, noradrenaline, thyrotropin-releasing hormone, corticotropin-releasing hormone, insulin, glucagon, gastrin, secretin, motilin, and cholecystokinin (Gomes-Porras et al., 2020; Patel, 1999; Zhang et al., 2025). It plays vital roles in cognitive functions, learning, and memory (Borbély et al., 2013). Besides its endocrine effects, it also suppresses vascular, immune, neuronal functions, and cell proliferation (Günther et al., 2018; Patel, 1999). SRIF-14 signals through five G-protein-coupled receptors, SST_1-5_ (Theodoropoulou and Stalla, 2013), which are classified into two subfamilies based on their synthetic agonist-binding selectivity: SRIF1 (SST_2,3,5_) and SRIF2 (SST_1,4_) (Patel, 1999). SRIF1 receptors are involved in endocrine effects, while SRIF2 receptors are linked to analgesic and anti-inflammatory actions (Helyes et al., 2001). Its strong antisecretory properties (Theodoropoulou and Stalla, 2013) have made SRIF-14 important in treating Alzheimer’s, Cushing’s disease, type 2 diabetes, neuroendocrine tumors, acromegaly, pain-related conditions (inflammation, neuropathy, rheumatoid arthritis), and depression (Boehm and Lustig, 2002; Chanson, 2016; Hofland, 2008; Rai et al., 2015; Sun & H. Coy, 2016). However, its clinical application is limited by its short half-life and broad actions. Its anti-inflammatory and antihyperalgesic effects are mainly mediated via SST_4_, without endocrine effects, as shown in various animal models (Helyes et al., 2004; Pintér et al., 2002; 2006; 2014; Szolcsányi et al., 2004). Therefore, SST_4_ is a promising target for developing new anti-inflammatory and analgesic drugs, emphasizing the need to create and characterize metabolically stable and selective agonists.

**FIGURE 1:**
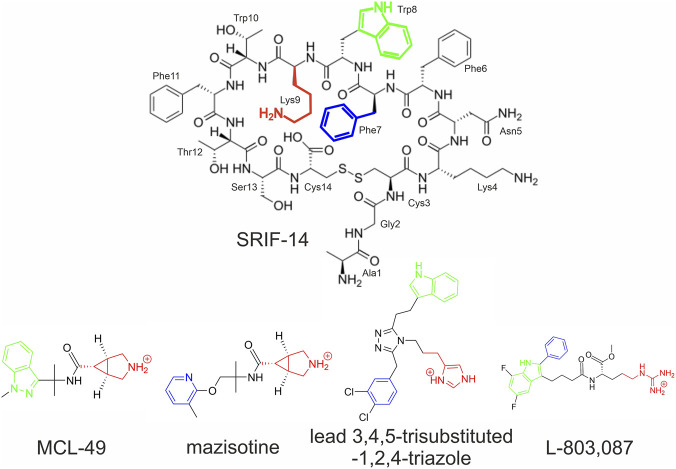
The Lewis structures of SRIF-14 and the investigated compounds. The phenyl and indole rings mimicking Phe7 and Trp8 of SRIF-14 are coloured blue and green, respectively. The protonated nitrogen-containing molecular components resembling the Lys9 residue of SRIF-14 are highlighted in red.

The investigated compounds (Figure 1) were selected to demonstrate that structurally diverse synthetic SST_4_ receptor agonists can exert anti-hyperalgesic effects. Additionally, all compounds show structural similarity to the β-turn pharmacophore region of SRIF-14. The protonated N atom, the indole, and the phenyl/pyridine ring were designed to mimic Lys9, Trp8, and Phe7 of SRIF-14, respectively. Lys9 of SRIF-14 forms a salt-bridge interaction with the conserved Asp residue in transmembrane (TM) region III of all SST receptors, which plays a key role in ligand binding and receptor activation (Zhao et al., 2022). Trp8 of SRIF-14 is essential for SST_4_ activation and pharmacological effects. Phe7 of SRIF-14 is surrounded by a hydrophobic pocket formed by extracellular loop 2 (ECL2) and helix V, and substitution of Phe7 with mesityl-alanine can cause a spatial collision in this hydrophobic pocket and significantly affect binding (Zhao et al., 2022).

MCL-49 and mazisotine were initially patented by Boehringer Ingelheim (Slater and Kontoyianni, 2022). Mazisotine is currently available for research purposes, and Eli Lilly has evaluated its therapeutic potential in a Phase II clinical trial for diabetic neuropathy and osteoarthritis (Borbély and Pethő, 2024). It was selected because it had reached clinical trials and lacked published *in vivo* mouse data. MCL-49, a related compound, was chosen to assess whether mazisotine offers advantages within the same compound family. The lead 3,4,5-trisubstituted-1,2,4-triazole molecule was designed as a selective, high-affinity SST_4_ receptor agonist (Daryaei et al., 2018). In silico docking, *in vitro* radioligand binding displacement studies, and a forskolin-induced cAMP inhibition assay have shown that this compound binds to and activates the SST_4_ receptor (Daryaei et al., 2018). Several compounds have also been developed by chemical modification of this lead triazole and have undergone comprehensive preclinical testing for the treatment of Alzheimer’s disease (Neumann et al., 2021). However, pharmacokinetic properties have not been predicted, and *in vivo* effects of the lead 3,4,5-trisubstituted-1,2,4-triazole have not been studied. L-803,087 is a commercially available, potent, and selective SST_4_ receptor agonist for experimental use. We also included these compounds, both structurally distinct SST_4_ receptor agonists, to compare their effects in a neuropathic pain model.

J-2156, the reference in this study, is a highly selective non-peptide SST_4_ receptor agonist that showed anti-nociceptive and anti-inflammatory effects in several acute and chronic animal models (Helyes et al., 2006; Park et al., 2019; Sándor et al., 2006).

In this paper, we characterize and compare these four SST_4_ agonists based on drug-likeness and pharmacokinetic predictions, as well as their binding properties *in silico*, SST_4_ receptor-linked cAMP inhibition *in vitro*, and antihyperalgesic effects in a traumatic neuropathy mouse model *in vivo*.

## 2 Methods

### 2.1 Materials

J-2156 (Cat. No.:6201, Tocris Bioscience), mazisotine (Cat.No.:HY-139347, MedChemExpress) and L-803,087 (Cat.No.:1979, Tocris Bioscience) were obtained from commercial sources. MCL-49 was provided by AVICOR Ltd. The lead 3,4,5-trisubstituted-1,2,4-triazole molecule was provided by A.M. Crider Department of Pharmaceutical Sciences, School of Pharmacy, Southern Illinois University Edwardsville) and synthesized by a previously described method (Daryaei et al., 2018).

CHO-K1 cells expressing the human SST_2_ or SST_4_ receptor (Eurofins DiscoverX, Fremont, CA, United States, RRID: CVCL_KV83) were used in the *in vitro* studies.

### 2.2 *In silico* modelling studies

#### 2.2.1 Drug-likeness and pharmacokinetic prediction

Data for the characterization of ligands, including molecular weight (MW), the logarithm of the octanol/water partition coefficient (logP_o/w_), the number of H-donor and H-acceptor atoms in the molecules, and CNS activity were predicted using the Schrödinger (2024).

#### 2.2.2 Target preparation

Crystal structure of SST_4_ was obtained from the Protein Data Bank (Berman, 2000) (PDB code: 7XMT (Zhao et al., 2022). Missing side chains and loops were built by SWISS-MODEL (Waterhouse et al., 2018). Further receptor preparation steps, including the addition of polar hydrogens, optimization of hydrogen-bond assignments, and restrained minimization of the receptor structure, were performed using the Protein Preparation Wizard module of Maestro (Madhavi Sastry et al., 2013). Restrained minimization means that the root mean squared deviation (RMSD) of the heavy atoms was set to 0.3 Å from the original. This optimized receptor structure was used as a target in the docking calculations.

#### 2.2.3 Ligand preparation

All ligands were built with Avogadro 1.2.0 (Hanwell et al., 2012). then minimized by MOPAC, a semi-empirical quantum chemistry program package (Moussa and Stewart, 2026) with a PM7 parametrization (Stewart, 2013). The gradient norm was set to 0.001. Force calculations were applied to the energy-minimized structures. These energy-minimized structures were used for docking calculations.

#### 2.2.4 Docking calculations

Docking of all ligands was performed using the Schrödinger Glide Ligand Docking Wizard (Yang et al., 2021), focusing on the extracellular region of SST_4_. Transmembrane and intracellular target regions were excluded from the docking search area to reduce false-positive conformations. Flexibility was allowed at all active torsions of the ligand, but the target was treated rigidly. The grid maps were prepared by the Receptor Grid Generation Wizard of Schrödinger (Friesner et al., 2004). The docking box was centered on the extracellular region of SST_4,_ including 30 × 26 × 26 grid points at a 2.000 Å spacing. Ten poses of each ligand were written out, which were ranked by the Ligand Docking Wizard automatically. Rank one of each ligand was selected as a representative structure and used for further analysis.

### 2.3 *In vitro* cAMP inhibition assay

SST_4_ is a G protein-coupled receptor that inhibits adenylate cyclase activity and cAMP formation. Forskolin is a cell-permeable diterpene that directly activates adenylate cyclase and increases intracellular cAMP levels (Alasbahi and Melzig, 2012). In SST_2_- or SST_4_-expressing CHO cells, the ability of the compounds to reduce cAMP levels was investigated after forskolin pretreatment using a luminescence assay.

CHO-K1 cells expressing the human SST_2_ or SST_4_ receptor (Eurofins DiscoverX, Fremont, CA, United States, RRID: CVCL_KV83) were cultured in Dulbecco’s Modified Eagle’s Medium/Nutrient Mixture F-12 Ham (DMEM/F12, Thermo Fisher Scientific, United States) and kept at 37 °C, 5% CO_2_ incubator until 70%–80% confluence. The media was supplemented with 2 mM L-glutamine (Thermo Fischer Scientific, United States), 10% fetal bovine serum (FBS), 1x penicillin/streptomycin (Thermo Fischer Scientific, United States), and 800 μg/mL selection antibiotic G418 (Eurofins DiscoverX, Fremont, CA, United States). The passage number of SST_2_- or SST_4_-expressing CHO cells was 4. The cAMP level was measured using the DiscoverX HitHunter™ cAMP assay kit (Eurofins DiscoverX, Fremont, CA, United States). 10 mM stock solutions were prepared in dimethyl sulfoxide (DMSO, Sigma-Aldrich Ltd., Hungary, 67–68–5) and kept at −20 °C until future use. Cells were seeded into a white 96-well assay plate in 100 μL cell plating reagent (Eurofins DiscoverX, Fremont, CA, United States) at a density of 20,000 cells/well and incubated overnight at 37 °C, 5% CO_2_. The next day, the cell plating reagent (Eurofins DiscoverX, Fremont, CA, United States) was aspirated and replaced with PBS. A series of serial dilutions (10^–11^ M–10^–5^ M) of compounds with PBS containing the phosphodiesterase inhibitor rolipram (Sigma-Aldrich Ltd., Hungary, 61413–54–5) and the adenylate cyclase stimulator forskolin (Sigma-Aldrich Ltd., Hungary, 66575–29–9) (100 μmol) was performed. Then, cells were treated with different concentrations of the SST_4_ ligands for 30 min at 37 °C, each in duplicates. Once ligand treatment was completed, each following step involved incubations with the assay reagents (Eurofins DiscoverX, Fremont, CA, United States) at room temperature. The chemiluminescent signal corresponding to the cAMP concentration was detected using a PerkinElmer EnSpire Alpha plate reader. The data were expressed as cAMP accumulation as the percentage of the forskolin response. The data obtained from the measurements were normalized to simplify comparison by defining 0% and 100% as the average responses of Rolipram- and Forskolin-treated wells (n = 4 per biological replicate), respectively (Wang et al., 2017; Hoare et al., 2020). GraphPad Prism version 8.0.1 for Windows (GraphPad Software, San Diego, CA, United States) was used for graphs and calculations.

In each experiment, the reference compounds J-2156 and octreotide (data not shown) were included on every assay plate in the same concentration range as the test compounds and served as internal controls (Bo et al., 2022; Shenoy et al., 2018). These reference ligands were consistently used to verify assay performance and receptor responsiveness. Based on extensive prior measurements, their concentration–response profiles are highly reproducible across independent experiments; therefore, their inclusion ensured the reliability of the model system and supported the validity of the results obtained for the investigated compounds.

### 2.4 *In vivo* experiments

#### 2.4.1 Animals

*In vivo* experiments were conducted on male NMRI mice (12–14 weeks old, 28–35 g). The original breeding pairs were purchased from Jackson Laboratories (United States) via Animalab Hungary. To standardize the model and exclude variability caused by the estrous cycle of female animals, and to comply with the principle of reducing the number of animals used in the study, investigations were performed exclusively on male mice. Animals were bred and housed in a temperature-controlled (21 °C–23 °C) and humidity-controlled (50%–70%) environment with a 12-12 h light-dark cycle (lights on at 6:00 a.m.) in groups of three to five in polycarbonate cages (365 × 207 × 140 mm) at the animal facilities of the Department of Pharmacology and Pharmacotherapy, University of Pécs, provided with rodent chow and water *ad libitum*.

#### 2.4.2 Ethics

All experimental procedures were conducted in accordance with European legislation (Directive 2010/63/EU) and Hungarian Government regulation (40/2013., II. 14.) on the protection of animals used for scientific purposes. All procedures were approved by the Ethics Committee on Animal Research of the University of Pécs in accordance with the Ethical Codex of Animal Experiments; a license was granted (BA02/2000-15/2023). All efforts were made to minimize the number of animals used and their suffering during the experiments. This study was prepared according to the ARRIVE 2.0 (Percie du Sert et al., 2020). The investigator who measured the mechanical pain threshold values was blinded to the drug treatment of the different investigational groups.

#### 2.4.3 Sciatic-nerve ligation-induced traumatic neuropathy model

The partial sciatic nerve ligation model (PSNL) is a well-established and widely accepted model of neuropathic pain (Colleoni and Sacerdote, 2010) that was initially described in rats but was later modified for mice (Sándor et al., 2006). The procedure involves the tight ligation of thinly myelinated and unmyelinated fibers, inducing abnormal sensory function, such as mechanical hyperalgesia, which refers to chronic neuropathic pain. All surgical procedures were performed under ketamine-xylazine anesthesia (100/5 mg/kg i. p.). The unilateral sciatic nerve was exposed, and 1/3–1/2 of the nerve trunk was tightly ligated with a silicone silk suture (Ethicon 8–0). The wound was closed with interrupted stitches using 4–0 nylon suture thread, and the animals were allowed to survive.

#### 2.4.4 Measuring the mechanonociceptive threshold of the paw

The baseline values of the mechanonociceptive thresholds were determined on two consecutive days using dynamic plantar aesthesiometry (DPA, Ugo Basile, Comerio, Italy), which functions as an electric von-Frey device (Szabó et al., 2005; Tékus et al., 2014). Mice were housed in individual testing boxes with a metal mesh floor. Increasing force (0–10 g) was applied with a blunt-end needle to the plantar surface of hindpaws until the paw withdrawal response was elicited. Actual force value measured at the moment of paw withdrawal was recorded as the mechanonociceptive threshold. Seven days after PSNL, mechanonociceptive thresholds of the hindpaws were measured before the SST_4_ agonist treatments, as well as 30 and 60 min afterward. Mechanical hyperalgesia was determined as a percentage change of the baseline threshold values at these time points, while the anti-hyperalgesic effect was calculated as follows ((pretreatment hyperalgesia–posttreatment hyperalgesia)/pretreatment hyperalgesia x 100).

#### 2.4.5 Administration of the SST_4_ agonists

Seven days after surgery, mice were randomly assigned to the different treatment groups to ensure equal average postoperative mechanical hyperalgesia across groups. Animals with less than 20% pre-treatment mechanical hyperalgesia were excluded from the study. Each drug candidate was administered intraperitoneally (i.p.) at three doses (0.01, 0.3, and 3 mg/kg) in 3% DMSO-saline. The mechanonociceptive threshold was determined 30 and 60 min after drug administration to assess analgesic effects.

### 2.5 Data and statistical analysis

GraphPad Prism version 8.0.1 (GraphPad Software, San Diego, CA, United States) was used for *in vitro* graphs and calculations. Results are expressed as means ± SEM. cAMP levels were normalized to the forskolin response, set at 100%. Two independent biological experiments in technical replicates (n = 4) were used to ensure the reliability of single values. Curves were fit by nonlinear regression using the sigmoidal dose–response equation.

*In vivo* results were statistically analyzed using GraphPad Prism 9 software (GraphPad Software, San Diego, CA, United States). Data are expressed as the mean ± SEM for n = 6-9 mice per group. Because statistical significance and non-significance do not always reflect biological relevance or equivalence, effect sizes were calculated using Hedges’ g (difference in means divided by the pooled and weighted standard deviation). These analyses were used to evaluate and visualize the data. An effect size >0.2 was considered small, >0.5 was defined as medium, and >0.8 was considered large (Cohen, 1992). Data were also analyzed using a two-way ANOVA followed by Bonferroni’s multiple-comparison test, with *p < 0.05 considered statistically significant. These calculations are reported in the Supplementary material.

The data and statistical analysis comply with the recommendations on experimental design and analysis in pharmacology.

## 3 Results

### 3.1 *In silico* results

#### 3.1.1 All compounds satisfy drug-likeness requirements

The drug-likeness properties, including violations of Lipinski’s Rule of Five (ROF) and pharmacokinetic parameters, were predicted using an *in silico* approach (Table 1). All compounds complied with Lipinski’s ROF, except for a few points (Table 1). The logP_o/w_ value of the lead 3,4,5-trisubstituted-1,2,4-triazole exceeds 5, indicating poor water solubility. MCL-49 and mazisotine exhibit good CNS penetration, which is required for their central analgesic effects. However, this parameter is less promising for the lead 3,4,5-trisubstituted-1,2,4-triazole and L-803,087.

**TABLE 1:** Predicted Lipinski’s ROF descriptors, CNS penetration and interaction energies of the compounds.

Property	Lipinski’s ROF limits	MCL-49	Mazisotine	Lead 3,4,5-trisubstituted-1,2,4-triazole	L-803,087
MW^a^ (Da)	500	299.387	290.377	480.411	486.533
N_H-donors_ ^b^	5	2	2	2	5.25
N_H-acceptors_ ^b^	10	5	5	4	5.75
logP_o/w_ ^c^	5	1.983	2.037	6.617	3.635
CNS penetration^d^	-	1	1	−1	−2
E_inter_ (kcal*mol^-1^)^e^	-	−6.06	−4.81	−7.17	−7.62

^a^ molecular weight (MW).

^b^ number of hydrogen donors and hydrogen acceptors (N_H-donors_, N_H-acceptors_). For N_H-donors_ and N_H-acceptors_, the values are averages across several protonation states, resulting in non-integer values.

^c^ the logarithm of octanol/water partition coefficient (logP_o/w_).

^d^ CNS penetration reflects the central nervous system activity of the compounds and is reported on a −2 (inactive) to +2 (active) scale (Schrödinger Release 2024–3: Schrüdinger, 2024).

^e^ Interaction energy in kcal*mol^-1^.

#### 3.1.2 All compounds predicted to form a salt-bridge interaction with receptor Asp126 via their protonated nitrogen, based on docking results

**FIGURE 2:**
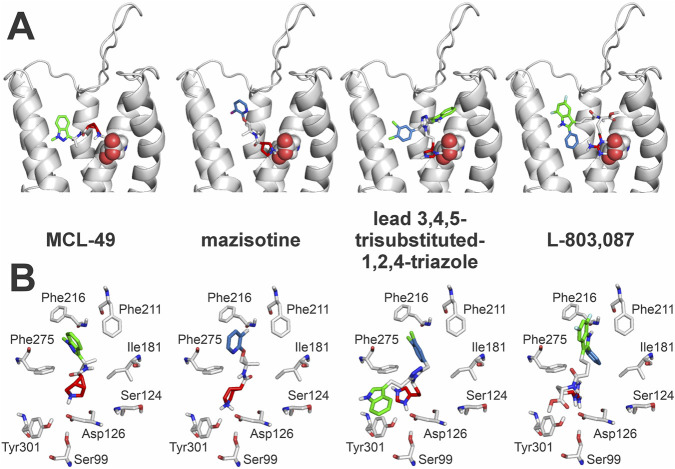
**(A)** A global view of the SST4 receptor (gray, cartoon representation) in complex with Rank 1 representative of the ligands. Mimicking groups for Phe7, Trp8, and Lys9 of SRIF-14 are highlighted with blue, green, and red, respectively. Asp126 of the SST4 receptor is highlighted with a sphere representation. **(B)** The close-up view of the investigated compounds (Rank 1 representative) in the SST4 receptor binding pocket. Target residues interacting with the ligands within 3.5 Å are coloured gray. Mimicking groups for Phe7, Trp8, and Lys9 of SRIF-14 are highlighted with blue, green, and red, respectively.

The docking calculations yielded a representative atomic-resolution model of four ligand structures bound to the SST_4_ receptor (Figure 2A). The docking approach was validated by redocking J-2156 to the experimental structure (PDB code: 7XMT (Zhao et al., 2022)). The RMSD between the experimental and docked J-2156 was 3.28 Å. The interaction energy was −7.00 kcal/mol.

Docking results showed that all compounds fit into a deep binding pocket of the SST_4_ receptor, accessible from the extracellular region and located in the crevice between the transmembrane (TM) helices three to seven and the ECL 2–3. All compounds predicted to form a salt-bridge interaction with receptor Asp126 via their protonated nitrogen (Figure 2A), as observed in the pharmacophores of SRIF-14 and J-2156 (Supplementary Table S1).

The close-up view shows the binding mode of the compounds, highlighting target residues within 3.5 Å of the ligand (Figure 2B; Supplementary Table S1). Phe275 and Tyr276 form hydrophobic interactions with the indole ring of MCL-49 and the phenyl ring of mazisotine, lead 3,4,5-trisubstituted-1,2,4-triazole, and L-803,087, as in the reference molecule, J-2156. The indole ring of the lead 3,4,5-trisubstituted-1,2,4-triazole occupies a hydrophobic sub-pocket formed by helices II, III, and ECL1, as does the phenyl ring of J-2156. All compounds bind to SST_4_ with similar interaction energies (E_inter_) (Table 1).

### 3.2 All compounds similarly inhibit SST_4_ activation-induced cAMP accumulation based on *in vitro* results

The *in vitro* receptor activation data confirmed the *in silico* binding results.

**FIGURE 3:**
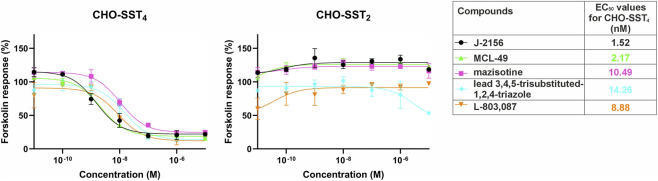
Concentration-response curves of the tested compounds, demonstrating their ability to suppress cAMP levels in SST_4_- or SST_2_-expressing CHO cells. EC_50_ values of the investigated compounds in comparison with J-2156 in SST_4_-expressing CHO cells in cAMP accumulation assay. Data were normalized by defining 0% and 100% as the mean responses of Rolipram- and Forskolin-treated wells, respectively. J-2156 was included as a reference superagonist. All data represent the mean ± SEM from two independent biological experiments conducted in technical duplicates (n = 4) for each compound. EC_50_ values were derived from normalized concentration–response curves by nonlinear regression analysis (log [inhibitor] vs. response, three-parameter model) using GraphPad Prism.

All compounds showed concentration-dependent inhibition in SST_4_-expressing CHO cells, with efficacies and potencies comparable to those of the selective SST_4_ agonist reference compound J-2156. The EC_50_ values are similar for mazisotine, lead 3,4,5-trisubstituted-1,2,4-triazole, and L-803,087, but MCL-49 is more potent than the other compounds (Figure 3). None of the compounds showed a significant effect in SST_2_-expressing CHO cells (Figure 3).

### 3.3 All compounds inhibit traumatic neuropathic hyperalgesia

**FIGURE 4:**
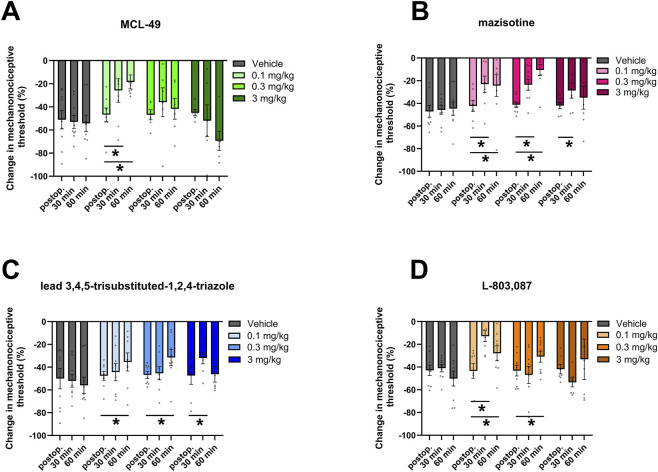
Mechanical hyperalgesia (decrease of the mechanonociceptive threshold in percentage change compared to the pre-operative value) of the operated hindpaws. This parameter was reversed by all test compounds: **(A)** MCL-49 was effective in the lowest dose, **(B)** mazisotine and **(C)** lead 3,4,5-trisubstituted-1,2,4-triazole were effective in all investigated doses, and **(D)** L-803,087 was effective in both doses of 0.1 and 0.3 mg/kg, depending on the examination time point. Each column represents the mean ± SEM of n = 6-9 mice per group and individual data points are represented by gray dots. Statistical analysis was performed using effect size analysis (*large effect size: Hedge’s g ≥ 0.8).

One week after PSNL, a 40%–50% decrease in the mechanonociceptive threshold was observed in the operated right hindpaw across all groups, indicating the development of mechanical hyperalgesia (Figures 4A–D; Supplementary Table S2). Raw mechanonociceptive threshold data are shown in Supplementary Table S3. This mechanical hyperalgesia was not influenced by any vehicle treatments, at either 30 or 60 min after administration. At both examined time points, 0.1 mg/kg MCL-49 increased the mechanonociceptive threshold with a large effect size (Figure 4A). For mazisotine, the 0.1 mg/kg and 0.3 mg/kg doses elevated the mechanonociceptive threshold with a large effect size at both time points. In contrast, the 3 mg/kg dose of mazisotine increased the mechanical pain threshold with a large effect size at 30 min, but not at 60 min (Figure 4B). Interestingly, all treatments with the lead 3,4,5-trisubstituted-1,2,4-triazole increased the mechanonociceptive threshold with a large effect size: 0.1 and 0.3 mg/kg at 60 min, while 3 mg/kg at 30 min (Figure 4C). In case of L-803,087, the lower doses (0.1 and 0.3 mg/kg) induced an increase of the mechanical pain threshold with large effect size, however, the highest dose (3 mg/kg) showed no effect (Figure 4D). Effect size values and p values for the mechanical hyperalgesia measurements are summarized in Supplementary Tables S4, S54, respectively.

**FIGURE 5:**
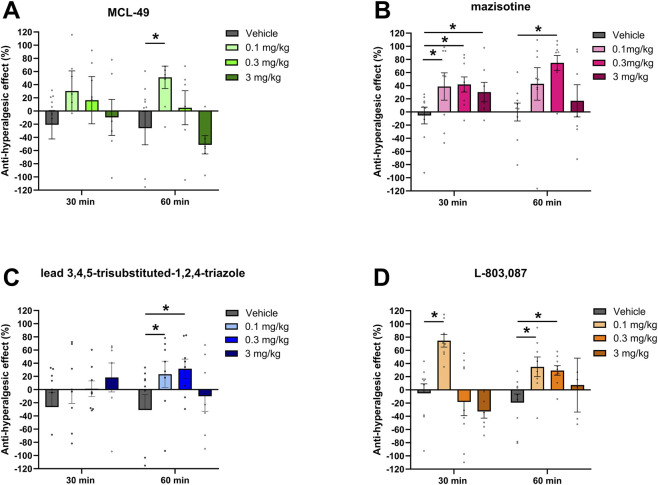
Anti-hyperalgesic effect of the test compounds was calculated as follows ((pretreatment hyperalgesia-posttreatment hyperalgesia)/pretreatment hyperalgesia × 100) and compared to the vehicle-treated group 30 min and 60 min after i.p. treatment in mice with partial sciatic nerve ligation. **(A)** MCL-49, **(B)** mazisotine, **(C)** lead 3,4,5-trisubstituted-1,2,4-triazole, and **(D)** L-803,087 exhibited significant anti-hyperalgesic effects that varied by dose and time point. Each column represents the mean ± SEM of n = 6-9 mice per group, with individual data points shown as gray dots. Statistical analysis was performed using effect size analysis (*large effect size: Hedge’s g ≥ 0.8).

Based on the pre- and posttreatment hyperalgesia values, anti-hyperalgesic effects of all compounds were also calculated (Figures 5A–D; Supplementary Table S6). The lowest dose (0.1 mg/kg) of MCL-49 showed an anti-hyperalgesic effect of ca. 50% 60 min post-treatment, with a large effect size (Figure 5A). All doses of mazisotine improved mechanical hyperalgesia 30 min following treatment, with effects ranging from 30%–42% compared with the vehicle group, also with a large effect size. The anti-hyperalgesic effect of the 0.3 mg/kg dose was also observable 60 min after treatment and amounted to ca. 75%, with a large effect size (Figure 5B). The lead 3,4,5-trisubstituted-1,2,4-triazole exerted an anti-hyperalgesic effect of 23%–32% 60 min after administration of the 0.1 and 0.3 mg/kg doses, with a large effect size (Figure 5C). For L-803,087, 0.1 mg/kg showed ca. 75% anti-hyperalgesic effect after 30 min, with a large effect size. The anti-hyperalgesic effect was around 30%–35% 60 min after treatment with the two lower doses, with a large effect size (Figure 5D). Effect size values and p values for the anti-hyperalgesic effect calculations are summarized in Supplementary Tables S7, S8, respectively.

## 4 Discussion

Our team has previously demonstrated that somatostatin released from the capsaicin-sensitive sensory nerve terminals mediates antinociceptive effects via the SST_4_ receptor (Helyes et al., 2004; 2006; Pintér et al., 2006; Sándor et al., 2006; Szolcsányi et al., 1998). It was also previously shown that J-2156, a highly selective SST_4_ agonist, inhibits the release of sensory neuropeptides, including substance P, calcitonin gene-related peptide, and somatostatin (Helyes et al., 2006). Additionally, it mediates analgesic effects in both acute and chronic pain models in rats (Sándor et al., 2006). TT-232 is a heptapeptide somatostatin analogue, which is a high-affinity SST_4_ agonist. Our research group described the binding mechanism of this compound and demonstrated its analgesic and anti-inflammatory effects mediated by SST_4_ receptor activation (Börzsei et al., 2023; Helyes et al., 2004; Pintér et al., 2002). These data suggest that the SST_4_ receptor plays an important role in the reduction of pain and inflammation.

In this study, we conducted a comprehensive comparative evaluation of four synthetic SST_4_ receptor agonists. Our examinations included *in silico* predictions of drug-likeness, CNS penetration, and binding characteristics, *in vitro* SST_4_ receptor activation, and *in vivo* anti-hyperalgesic efficacy measurements in a mouse model of traumatic neuropathy.

All compounds met Lipinski’s ROF, except for a few points. Lipinski’s ROF assesses whether a compound with pharmacological activity can become an orally active drug by analyzing specific molecular traits (Lipinski, 2004; Lipinski et al., 2012). In addition to Lipinski descriptors, *in silico* results indicated CNS penetration for MCL-49 and mazisotine, but it was less promising for 3,4,5-trisubstituted-1,2,4-triazole and L-803,087. It was previously shown that SST_4_ receptors are present in brain regions involved in pain processing and on primary sensory neurons (Kecskés et al., 2020). Therefore, the central analgesic effects of several compounds targeting SST4 receptors may contribute to pain relief.

Docking calculations showed that all compounds occupy the deep binding pocket of SST_4_, exhibiting binding patterns and interaction energies comparable to those of the J-2156 superagonist (Table 1). All compounds formed hydrophobic interactions with Phe275 and Tyr276 of SST_4_. A study of alanine mutations showed that interactions with these amino acids are crucial for ligand-induced receptor activation (Zhao et al., 2022).

The protonated nitrogen of the compounds predicted to form a salt-bridge interaction with receptor Asp126, as in Lys9 of SRIF-14 (Figure 2A). Asp126 belongs to the conserved amino acids on TM3 region of SSTRs. The salt-bridge interaction with this conserved aspartate has been shown by numerous mutational, functional, and structural experiments to play a critical role in ligand binding and receptor activation (Bo et al., 2022; Börzsei et al., 2022; Chen et al., 1999; Chen et al., 2022; Crider et al., 2024; Greenwood et al., 1997; Heo et al., 2022; Kaupmann et al., 1995; Liapakis et al., 1996; Nehrung et al., 1995; Robertson et al., 2022a; Robertson et al., 2022b; Zhao et al., 2022). For SRIF-14 and J-2156, mutation of the receptor Asp126 dramatically reduces SST_4_ signaling, supporting that the interaction between the protonated nitrogen and Asp126 is essential for receptor activation (Zhao et al., 2022).

The *in silico* binding data were confirmed by the *in vitro* receptor activation assay. All four compounds inhibited cAMP accumulation in SST_4_-overexpressing CHO cells in a concentration-dependent manner with efficacies comparable to those of the selective SST_4_ agonist reference compound J-2156. Based on the EC_50_ values, the potency of MCL-49 is similar to that of J-2156 and higher than that of the other compounds. However, in a previous study (Daryaei et al., 2018), the EC_50_ of the lead 3,4,5-trisubstituted-1,2,4-triazole was 6.8 nM, compared with 14.26 nM determined in our assay. None of the compounds showed a significant effect in SST_2_-expressing CHO cells, indicating a preference for SST_4_ over SST_2_.

Partial sciatic nerve ligation results in behavioral, histological, and molecular changes mimicking the key components of traumatic neuropathic pain. The model is suitable for reproducing characteristic features such as allodynia, hyperalgesia, and spontaneous pain, providing a relevant platform to study the underlying mechanisms and the efficacy of novel therapeutic interventions (Lindenlaub and Sommer, 2000; Zhou et al., 2025). Our *in vivo* studies have shown that all investigated small-molecule SST_4_ receptor agonists could alleviate neuropathic mechanical hyperalgesia. MCL-49 was most effective at the lowest dose, particularly 60 min after i.p. administration. Mazisotine showed an antihyperalgesic effect within 30 min post-treatment across all applied doses. The lead 3,4,5-trisubstituted-1,2,4-triazole and L-803,087 reached their therapeutic effect mainly 60 min after the i.p. injections.

*In silico* predictions suggested that only MCL-49 and mazisotine could cross the blood-brain barrier (BBB). Despite the poor or negligible brain penetration of the lead 3,4,5-trisubstituted-1,2,4-triazole and L-803,087, these compounds produced high pain-relieving effects. A similar finding was observed in our previous study, in which predicted CNS penetration did not correlate with pain-relieving effects (Szőke et al., 2020), suggesting substantial peripheral mechanisms.

It is important to note that *Sstr4* mRNA is expressed by primary sensory afferents of the dorsal root ganglia (DRG) and trigeminal ganglia (TRG), including peripheral nerve terminals (Kántás et al., 2021). The activation of SST_4_ leads to hyperpolarization of the peripheral sensory neuron and consequent inhibition of pain transmission. Therefore, all SST_4_ receptor agonists might exert their effects at the level of primary sensory neurons without the need to penetrate the CNS (Bjørn-Yoshimoto et al., 2025).

Besides, *Sstr4* has also been found in the spinal dorsal horn and in several pain-related brain regions (Kecskés et al., 2020), indicating that SST_4_ agonists, being able to cross the BBB, can interact with various distinct neuronal circuits of the pain pathway. Moreover, a neuropathic condition might alter BBB permeability, thereby altering the brain’s accessibility for the compounds (DosSantos et al., 2014).

Furthermore, most molecules showed no classical dose-dependent action in our *in vivo* studies, however, this is not surprising since SST_4_ activation also inhibits the release of endogenous inhibitory mediators (e.g., somatostatin itself) from the nerve endings of the primary sensory neurons, which might contribute to the development of non-linear dose-response relationships (Helyes et al., 2006), although the effect of dual signaling or potential off-target mechanisms cannot be excluded. Additionally, desensitization of the receptor might be an explanation for this phenomenon (Jung et al., 2006; Oomen et al., 2002; Smalley et al., 1998). Our findings are consistent with a previous study, in which other small-molecule SST_4_ agonists showed bell-shaped dose-response curves with significant anti-hyperalgesic effects achieved in the same dose range as in this study, further confirming our observations (Helyes et al., 2006).

These data point out that the analgesic effect of small-molecule SST_4_ agonists can involve both peripheral and central mechanisms, as well as complex interactions with several other neuronal mediators, thus explaining both the efficacy independent from the CNS penetration and the non-classical dose-response relationship.

SST_4_ is a promising target for peripheral and central pain modulation. Preclinical studies with selective SST_4_ agonists consistently demonstrate analgesic effects via peripheral and central mechanisms (Adamcyzk et al., 2022; Helyes et al., 2006; Kántás et al., 2019; Sándor et al., 2006). J-2156 has shown robust analgesic effects across various rodent models, including breast cancer-induced bone pain, acute pain models such as the formalin test, and chronic pain models such as carrageenan-induced paw inflammation (Helyes et al., 2006), Complete Freund’s adjuvant (CFA)-induced chronic inflammation, and neuropathic pain models (Börzsei et al., 2023; Kántás et al., 2021). J-2156 was also effective in streptozotocin-induced diabetic neuropathy, performing comparably to gabapentin and morphine in early-phase assessment (Kuo et al., 2024) and in chronic low back pain model, where J-2156 effectively reduced both primary and secondary hyperalgesia at lumbar segments (Park et al., 2019). Since approximately 30%–40% of tumor patients suffer from neuropathic cancer pain (Yoon and Oh, 2018), somatostatin receptor agonism might also be beneficial in the analgesic treatment of neuropathic states associated with neoplasias, such as hepatocellular carcinoma, gastric-type lymphoma, breast cancer, *etc.* (Milewska-Kranc et al., 2023; Periferakis et al., 2021; 2024).

The role of L-803,087 in pain mechanisms and the modulation of stress-related neuroendocrine activity has already been comprehensively clarified (Adamcyzk et al., 2022). Intrahippocampal administration in mice decreased both plasma and hippocampal corticosterone levels following footshock stress, demonstrating that SST_4_ activation can modulate the HPA axis. Furthermore, in models sensitized by stress, L-803,087 restored normal aversive behaviors and stress responses, highlighting its potential to modulate affective components of pain (Adamcyzk et al., 2022).

The small-molecule oral SST_4_ agonist mazisotine, developed by Eli Lilly, was originally patented by Boehringer Ingelheim (Slater and Kontoyianni, 2022). Although preclinical pain data in rodents are limited or not available due to confidentiality issues, this compound was investigated in Phase II clinical trials for osteoarthritis and chronic low back pain (NCT04627038, NCT04874636) (Borbély and Pethő, 2024). Its effectiveness in other indications, such as diabetic peripheral neuropathic pain, was also evaluated in clinical trials (NCT06074562, NCT04707157), (Borbély and Pethő, 2024). In summer 2025, Eli Lilly officially shelved mazisotine, its experimental SST_4_ agonist pain drug, and removed it from its research pipeline. Mazisotine showed some positive signals but failed to deliver consistent, robust analgesic efficacy across broader Phase II studies. Despite these facts, it can be assumed that SST_4_ may still be a promising drug target in neuropathic pain because it is biologically well-validated and modulates neuronal hyperexcitability, as well as neurogenic inflammation. The clinical failure of mazisotine likely reflects patient heterogeneity, modest monotherapy efficacy, and suboptimal trial design rather than a fundamental flaw of the target itself. SST_4_ is therefore most likely to be effective in carefully selected patients, at earlier disease stages, or as part of combination therapies.

This study serves as a comprehensive proof of concept for the anti-hyperalgesic activity of structurally diverse synthetic SST_4_ receptor agonists, demonstrating that varying chemical structures can effectively target the receptor to alleviate neuropathic pain in mice. A major strength of the research lies in addressing the significant unmet clinical need for non-opioid chronic pain management. Although these molecules are not yet optimized leads, they represent a critical starting point for the selection and optimization of novel leads, with the potential to provide potent analgesia without the side effects associated with broader somatostatin receptor activation.

The study is limited by a lack of extensive pharmacokinetic characterization and detailed intracellular signaling experiments. However, these aspects were beyond its primary scope, as the tested compounds are not yet designated as clinical leads. Furthermore, while the mouse model of partial sciatic nerve ligation is translationally relevant, future research must bridge the gap between animal results and human application. Clinically, these findings suggest that selective SST_4_ agonists could be combined with existing pharmaceutical agents targeting other SSTR subtypes—such as those used in oncology—to manage pain synergistically without interfering with endocrine functions, offering a promising multimodal approach to complex pain conditions.

## Data Availability

The raw data supporting the conclusions of this article will be made available by the authors, without undue reservation.
