# Supplementary material for: Comparative evaluation for small molecule somatostatin 4 receptor agonists: in silico, in vitro, and in vivo approaches
Source: Front Pharmacol. 2026 Jun 15;17:1838782. doi: 10.3389/fphar.2026.1838782 (PMC13311364; doi:10.3389/fphar.2026.1838782)
Supplement: Supplementary file 1 [file Supplementaryfile1.docx]

**Supplementary materials**

|  | pharmacophore region of SRIF-14  (Phe7Trp8Lys9Thr10) | J-2156 | MCL-49 | mazisotine | lead 3,4,5-trisubstituted-1,2,4-triazole | L-803,087 |
| --- | --- | --- | --- | --- | --- | --- |
| Val103 |  | x |  |  |  |  |
| Ser106 |  |  |  |  | x |  |
| Leu123 |  | x | x |  |  |  |
| Asp126 | x | x | x | x | x | x |
| Met130 |  |  | x | x |  |  |
| Phe131 |  | x |  |  |  |  |
| Cys198 | x | x |  |  |  |  |
| Asn199 | x | x |  |  |  | x |
| Leu200 |  | x |  |  | x | x |
| Ser208 | x |  |  |  |  |  |
| Val212 |  |  |  |  |  | x |
| Thr215 | x | x |  |  |  | x |
| Phe275 |  | x |  | x | x | x |
| Tyr276 |  | x | x |  |  |  |
| Gln279 | x | x | x |  | x |  |
| Tyr301 |  |  |  | x | x |  |

**Suppl. Table 1.** Target residues interacting with representative docked ligand structures within 3.5 Å. The investigated compounds formed a salt-bridge interaction with receptor Asp126, which interaction have key role in the receptor activation is highlighted with gray.

**Suppl. Table 2.** Mechanical hyperalgesia (decrease of the mechanonociceptive threshold in percentage change compared to the pre-operative value) of the operated hindpaws before and 30 and 60 min after treatment with three different doses of the four test compounds (MCL-49, mazisotine, lead 3,4,5-trisubstituted-1,2,4-triazole, L-803,087). Data are expressed as mean ± SEM.

| **MCL-49** | **Vehicle** | **0.1 mg/kg** | **0.3 mg/kg** | **3 mg/kg** |
| --- | --- | --- | --- | --- |
| postoperative before treatment (%) | -51.10 ± 7.96 | -46.83 ± 5.91 | -46.90 ± 4.32 | -45.09 ± 2.69 |
| postoperative 30 min after treatment (%) | -52.98 ± 5.59 | -25.80 ± 10.15 | -35.99 ± 12.46 | -51.99 ± 13.82 |
| postoperative 60 min after treatment (%) | -54.29 ± 7.13 | -18.42 ± 6.06 | -41.67 ± 9.05 | -69.30 ± 8.23 |
| **Mazisotine** | **Vehicle** | **0.1 mg/kg** | **0.3 mg/kg** | **3 mg/kg** |
| postoperative before treatment (%) | -47.11 ± 5.03 | -42.32 ± 4.66 | -41.19 ± 2.42 | -41.93 ± 2.74 |
| postoperative 30 min after treatment (%) | -45.82 ± 3.74 | -23.04 ± 7.19 | -23.51 ± 4.83 | -28.66 ± 6.73 |
| postoperative 60 min after treatment (%) | -44.60± 5.82 | -24.26 ± 9.60 | -10.53 ± 4.88 | -34.94 ± 10.21 |
| **Lead 3,4,5-trisubstituted-1,2,4-triazole** | **Vehicle** | **0.1 mg/kg** | **0.3 mg/kg** | **3 mg/kg** |
| postoperative before treatment (%) | -50.01 ± 8.96 | -47.56 ± 3.90 | -46.77 ± 3.12 | -47.38 ± 7.67 |
| postoperative 30 min after treatment (%) | -52.07 ± 5.46 | -44.30 ± 7.48 | -45.34 ± 5.76 | -31.95 ± 5.08 |
| postoperative 60 min after treatment (%) | -55.99 ± 7.22 | -35.44 ± 7.95 | -31.58 ± 7.05 | -46.23 ± 6.91 |
| **L-803,087** | **Vehicle** | **0.1 mg/kg** | **0.3 mg/kg** | **3 mg/kg** |
| postoperative before treatment (%) | -42.91 ± 4.55 | -43.45 ± 6.46 | -43.24 ± 4.66 | -41.85 ± 4.22 |
| postoperative 30 min after treatment (%) | -41.02 ± 3.23 | -12.84 ± 4.78 | -46.89 ± 7.62 | -53.49 ± 3.98 |
| postoperative 60 min after treatment (%) | -50.15 ± 6.57 | -27.86 ± 6.61 | -30.89 ± 4.86 | -33.21 ± 17.86 |

**Suppl. Table 3.** Average values of the mechanonociceptive threshold of the operated hindpaws in grams. Data are expressed as mean±SEM.

| **MCL-49** | **Vehicle** | **0.1 mg/kg** | **0.3 mg/kg** | **3 mg/kg** |
| --- | --- | --- | --- | --- |
| baseline (g) | 9.1 ± 0.1 | 8.6 ± 0.3 | 8.9 ± 0.1 | 9.2 ± 0.1 |
| postoperative before treatment (g) | 4.4 ± 0.7 | 4.6 ± 0.6 | 4.8 ± 0.4 | 5.1 ± 0.2 |
| postoperative 30 min after treatment (g) | 4.2 ± 0.5 | 6.3 ± 0.8 | 5.7 ± 1.1 | 4.3 ± 1.2 |
| postoperative 60 min after treatment (g) | 4.1 ± 0.6 | 6.9 ± 0.3 | 5.2 ± 0.8 | 2.8 ± 0.7 |
| **Mazisotine** | **Vehicle** | **0.1 mg/kg** | **0.3 mg/kg** | **3 mg/kg** |
| baseline (g) | 9 ± 0.1 | 8.8 ± 0.2 | 9 ± 0.2 | 9.1 ± 0.1 |
| postoperative before treatment (g) | 4.8 ± 0.5 | 5.3 ± 0.4 | 5.3 ± 0.2 | 5.3 ± 0.3 |
| postoperative 30 min after treatment (g) | 4.9 ± 0.3 | 7.1 ± 0.7 | 6.8 ± 0.4 | 6.5 ± 0.6 |
| postoperative 60 min after treatment (g) | 5 ± 0.5 | 6.9 ± 0.9 | 8 ± 0.4 | 5.9 ± 0.9 |
| **Lead 3,4,5-trisubstituted-1,2,4-triazole** | **Vehicle** | **0.1 mg/kg** | **0.3 mg/kg** | **3 mg/kg** |
| baseline (g) | 8.9 ± 0.2 | 8.7 ± 0.3 | 9 ± 0.1 | 8.8 ± 0.1 |
| postoperative before treatment (g) | 4.4 ± 0.8 | 4.6 ± 0.4 | 4.8 ± 0.3 | 4.6 ± 0.6 |
| postoperative 30 min after treatment (g) | 4.2 ± 0.5 | 4.8 ± 0.6 | 4.9 ± 0.5 | 6 ± 0.5 |
| postoperative 60 min after treatment (g) | 3.9 ± 0.6 | 5.6 ± 0.7 | 6.2 ± 0.7 | 4.7 ± 0.6 |
| **L-803,087** | **Vehicle** | **0.1 mg/kg** | **0.3 mg/kg** | **3 mg/kg** |
| baseline (g) | 8.7 ± 0.3 | 8.8 ± 0.2 | 9 ± 0.2 | 9.1 ± 0.1 |
| postoperative before treatment (g) | 5 ± 0.5 | 4.9 ± 0.5 | 5.1 ± 0.4 | 5.3 ± 0.4 |
| postoperative 30 min after treatment (g) | 5.1 ± 0.3 | 7.6 ± 0.3 | 4.7 ± 0.7 | 4.2 ± 0.4 |
| postoperative 60 min after treatment (g) | 4.4 ± 0.6 | 6.4 ± 0.6 | 6.2 ± 0.5 | 4.1 ± 0.8 |

**Suppl. Table 4.** Effect size values referring to the changes of the mechanical hyperalgesia 30 and 60 min after treatment with three different doses of the four test compounds (MCL-49, mazisotine, lead 3,4,5-trisubstituted-1,2,4-triazole, L-803,087) in comparison with the postoperative control values. (Calculations have been made using Hedges’ formula.)

| **MCL-49** | | | | | | |
| --- | --- | --- | --- | --- | --- | --- |
|  | **0.1 mg/kg** | | **0.3 mg/kg** | | **3 mg/kg** | |
|  | 30 min | 60 min | 30 min | 60 min | 30 min | 60 min |
| Effect Size | 1.12 | 1.82 | 0.70 | 0.58 | 0.04 | -0.68 |
| **Mazisotine** | | | | | | |
|  | **0.1 mg/kg** | | **0.3 mg/kg** | | **3 mg/kg** | |
|  | 30 min | 60 min | 30 min | 60 min | 30 min | 60 min |
| Effect Size | 1.41 | 0.90 | 1.72 | 2.11 | 1.19 | 0.44 |
| **Lead 3,4,5-trisubstituted-1,2,4-triazole** | | | | | | |
|  | **0.1 mg/kg** | | **0.3 mg/kg** | | **3 mg/kg** | |
|  | 30 min | 60 min | 30 min | 60 min | 30 min | 60 min |
| Effect Size | 0.41 | 0.93 | 0.41 | 1.17 | 1.32 | 0.48 |
| **L-803,087** | | | | | | |
|  | **0.1 mg/kg** | | **0.3 mg/kg** | | **3 mg/kg** | |
|  | 30 min | 60 min | 30 min | 60 min | 30 min | 60 min |
| Effect Size | 2.42 | 1.16 | -0.33 | 1.11 | -1.24 | 0.49 |

**Suppl. Table 5.** Summary of the p values referring to the changes of the mechanical hyperalgesia 30 and 60 min after treatment with three different doses of the four test compounds (MCL-49, mazisotine, lead 3,4,5-trisubstituted-1,2,4-triazole, L-803,087) in comparison with the postoperative control values. (Two-way ANOVA followed by Bonferroni’s post hoc test.)

| **MCL-49** | | | | | | |
| --- | --- | --- | --- | --- | --- | --- |
|  | **0.1 mg/kg** | | **0.3 mg/kg** | | **3 mg/kg** | |
|  | 30 min | 60 min | 30 min | 60 min | 30 min | 60 min |
| P value | 0.3447 | 0.0554 | 0.9999 | 0.9999 | 0.9999 | 0.0336 |
| **Mazisotine** | | | | | | |
|  | **0.1 mg/kg** | | **0.3 mg/kg** | | **3 mg/kg** | |
|  | 30 min | 60 min | 30 min | 60 min | 30 min | 60 min |
| P value | 0.1461 | 0.3244 | 0.0273 | 0.0011 | 0.3482 | 0.9999 |
| **Lead 3,4,5-trisubstituted-1,2,4-triazole** | | | | | | |
|  | **0.1 mg/kg** | | **0.3 mg/kg** | | **3 mg/kg** | |
|  | 30 min | 60 min | 30 min | 60 min | 30 min | 60 min |
| P value | 0.9999 | 0.6009 | 0.9999 | 0.2049 | 0.5508 | 0.9999 |
| **L-803,087** | | | | | | |
|  | **0.1 mg/kg** | | **0.3 mg/kg** | | **3 mg/kg** | |
|  | 30 min | 60 min | 30 min | 60 min | 30 min | 60 min |
| P value | 0.0006 | 0.1307 | 0.9999 | 0.0127 | 0.0350 | 0.9999 |

**Suppl. Table 6.** Anti-hyperalgesic effect of three different doses of the four test compounds (MCL-49, mazisotine, lead 3,4,5-trisubstituted-1,2,4-triazole, L-803,087) 30 and 60 min after treatment. Data are expressed as mean ± SEM.

| **MCL-49** | **Vehicle** | **0.1 mg/kg** | **0.3 mg/kg** | **3 mg/kg** |
| --- | --- | --- | --- | --- |
| postoperative 30 min after treatment (%) | -20.76 ± 21.66 | 30.37 ± 30.67 | 16.43 ± 35.73 | -9.60 ± 27.23 |
| postoperative 60 min after treatment (%) | -25.95 ± 25.30 | 51.10 ± 17.13 | 5.00 ± 25.89 | -51.24 ± 13.91 |
| **Mazisotine** | **Vehicle** | **0.1 mg/kg** | **0.3 mg/kg** | **3 mg/kg** |
| postoperative 30 min after treatment (%) | -5.34 ± 12.72 | 38.78 ± 20.82 | 41.87 ±11.42 | 30.29 ± 14.77 |
| postoperative 60 min after treatment (%) | -0.12 ± 13.61 | 42.74 ± 24.72 | 74.75 ± 11.30 | 17.01 ± 24.46 |
| **Lead 3,4,5-trisubstituted-1,2,4-triazole** | **Vehicle** | **0.1 mg/kg** | **0.3 mg/kg** | **3 mg/kg** |
| postoperative 30 min after treatment (%) | -26.64 ± 22.47 | -0.75 ± 20.23 | 1.32 ± 11.78 | 18.4 ± 21.94 |
| postoperative 60 min after treatment (%) | -30.96 ± 23.68 | 23.26 ± 19.85 | 31.74 ± 14.49 | -10.09 ± 22.91 |
| **L-803,087** | **Vehicle** | **0.1 mg/kg** | **0.3 mg/kg** | **3 mg/kg** |
| postoperative 30 min after treatment (%) | -5.16 ± 14.33 | 74.60 ± 9.64 | -18.23 ± 20.83 | -32.66 ± 10.18 |
| postoperative 60 min after treatment (%) | -19.39 ± 13.08 | 34.97 ± 14.94 | 29.48 ± 7.27 | 7.31 ± 40.94 |

**Suppl. Table 7.** Effect size values referring to the changes of the anti-hyperalgesic effect 30 and 60 min after treatment with three different doses of the four test compounds (MCL-49, mazisotine, lead 3,4,5-trisubstituted-1,2,4-triazole, L-803,087) in comparison with the postoperative control values. (Calculations have been made using Hedges’ formula.)

| **MCL-49** | | | | | | |
| --- | --- | --- | --- | --- | --- | --- |
|  | **0.1 mg/kg** | | **0.3 mg/kg** | | **3 mg/kg** | |
|  | 30 min | 60 min | 30 min | 60 min | 30 min | 60 min |
| Effect Size | 0.67 | 1.19 | 0.50 | 0.43 | 0.17 | -0.40 |
| **Mazisotine** | | | | | | |
|  | **0.1 mg/kg** | | **0.3 mg/kg** | | **3 mg/kg** | |
|  | 30 min | 60 min | 30 min | 60 min | 30 min | 60 min |
| Effect Size | 0.90 | 0.76 | 1.30 | 2.00 | 0.92 | 0.33 |
| **Lead 3,4,5-trisubstituted-1,2,4-triazole** | | | | | | |
|  | **0.1 mg/kg** | | **0.3 mg/kg** | | **3 mg/kg** | |
|  | 30 min | 60 min | 30 min | 60 min | 30 min | 60 min |
| Effect Size | 0.41 | 0.84 | 0.52 | 1.06 | 0.71 | 0.31 |
| **L-803,087** | | | | | | |
|  | **0.1 mg/kg** | | **0.3 mg/kg** | | **3 mg/kg** | |
|  | 30 min | 60 min | 30 min | 60 min | 30 min | 60 min |
| Effect Size | 2.19 | 1.34 | -0.24 | 1.54 | -0.74 | 0.35 |

**Suppl. Table 8.** Summary of the p values referring to the antihyperalgesic effect of the three different doses of the four test compounds (MCL-49, mazisotine, lead 3,4,5-trisubstituted-1,2,4-triazole, L-803,087), in comparison with the vehicle-treated control group. (Two-way ANOVA followed by Bonferroni’s post hoc test.)

| **MCL-49** | | | | | | |
| --- | --- | --- | --- | --- | --- | --- |
|  | **0.1 mg/kg** | | **0.3 mg/kg** | | **3 mg/kg** | |
|  | 30 min | 60 min | 30 min | 60 min | 30 min | 60 min |
| P value | 0.7882 | 0.1497 | 0.9999 | 0.9999 | 0.9999 | 0.9999 |
| **Mazisotine** | | | | | | |
|  | **0.1 mg/kg** | | **0.3 mg/kg** | | **3 mg/kg** | |
|  | 30 min | 60 min | 30 min | 60 min | 30 min | 60 min |
| P value | 0.3967 | 0.4439 | 0.2601 | 0.0107 | 0.9008 | 0.9999 |
| **Lead 3,4,5-trisubstituted-1,2,4-triazole** | | | | | | |
|  | **0.1 mg/kg** | | **0.3 mg/kg** | | **3 mg/kg** | |
|  | 30 min | 60 min | 30 min | 60 min | 30 min | 60 min |
| P value | 0.9999 | 0.3375 | 0.9999 | 0.1687 | 0.7445 | 0.9999 |
| **L-803,087** | | | | | | |
|  | **0.1 mg/kg** | | **0.3 mg/kg** | | **3 mg/kg** | |
|  | 30 min | 60 min | 30 min | 60 min | 30 min | 60 min |
| P value | 0.0142 | 0.2060 | 0.9999 | 0.2957 | 0.9999 | 0.9999 |
